# Mapping internal and lateral anterior intercostal artery perforator flaps with colour Doppler ultrasound: correlation between preoperative imaging and intraoperative findings in oncoplastic breast surgery

**DOI:** 10.1007/s00404-026-08417-z

**Published:** 2026-04-09

**Authors:** Georg Schmidt, Theresa Mayo, Angela von Falkenhausen, Marion Kiechle, Daniel Müller

**Affiliations:** 1 Department of Gynecology and Obstetrics, TUM University Hospital and Comprehensive Cancer Center (CCCTUM), Technical University Munich, Munich, Germany; 2 Department of Gynecology and Obstetrics, University Hospital Mannheim, Heidelberg University, Mannheim, Germany

**Keywords:** Breast cancer, Oncoplastic surgery, ICAP flap, Chest wall perforator flap, Colour Doppler ultrasound, Perforator mapping

## Abstract

**Purpose:**

Anterior intercostal artery perforator (AICAP) flaps are valuable options for partial breast reconstruction, requiring reliable identification of dominant perforators for safe flap design. This study aimed to evaluate the agreement between preoperative high-frequency colour Doppler ultrasound (CDUS) findings and intraoperative anatomy of internal and lateral intercostal perforators, and to map their anatomical distribution.

**Methods:**

Sixty-four patients undergoing breast-conserving surgery were examined using high-frequency CDUS. The region from the midline to the mid-axillary line and from the fourth intercostal space to 4 cm below the inframammary fold was systematically scanned. Dominant perforators were measured, marked, and intraoperatively reassessed in 24 patients undergoing AICAP flap reconstruction. Vessel diameters, location, and additional perforators were recorded and spatial distribution was analysed.

**Results:**

Preoperative CDUS identified lateral perforators as larger than internal (2.08 mm vs. 1.61 mm, *p* < 0.01), confirmed intraoperatively (1.69 mm vs. 1.10 mm, p < 0.01). Dominant perforators were located 3.63 cm lateral to the patient’s midline and 1.32 cm inferior to the IMF (internal), and 0.53 cm medial to the anterior axillary line and 0.95 cm inferior to the IMF (lateral). In both internal and lateral regions, supplementary vessels were detected near the dominant perforators, contributing to increased perfusion security.

**Conclusions:**

High-frequency CDUS enables reproducible preoperative localisation of dominant IAICAP and LAICAP perforators and shows high agreement with intraoperative findings. Standardised ultrasound mapping may support structured flap planning and intraoperative orientation in oncoplastic breast reconstruction.

## What this study adds to clinical practice

**Table:** 

In this cohort, high-frequency colour duplex ultrasound (CDUS) enabled reproducible identification and localisation of anterior chest wall perforators. By reducing operative uncertainty during flap planning, this approach may facilitate broader clinical adoption of chest wall perforator flaps.
Furthermore, our work complements existing clinical research on CDUS in perforator flap planning by introducing a standardised ultrasound protocol, a coordinate-based mapping system, and a structured correlation between preoperative imaging and intraoperative findings, thereby strengthening the methodological framework for CDUS-guided perforator assessment.

## Introduction

Over the past three decades, the field of breast cancer surgery has undergone a paradigm shift from a purely oncological focus to a more holistic approach that integrates oncological safety with aesthetic and functional outcomes. This evolution has led to the emergence and refinement of oncoplastic breast surgery (OBS), a field that combines the principles of surgical oncology with plastic and reconstructive techniques. First introduced by Clough et al. in the late 1990s, OBS has enabled surgeons to perform more extensive tumour resections while preserving breast contour and symmetry in breast-conserving surgery [9].

As the demand for personalised and less invasive reconstructive techniques grew, so did the development of local tissue rearrangement and volume-replacement strategies. The introduction of perforator flaps has been a significant advance. These flaps, based on reliable perforator vessels, allow the transfer of well-vascularised fat and skin with minimal donor-site morbidity and without sacrificing the underlying muscle [18]. Over time, local perforator flaps, such as the (Anterior-, and Lateral-) Intercostal Artery Perforator, the Thoracodorsal Artery Perforator Flap (TDAP), or the LTAP flap (Lateral Thoracic Artery Perforator), have been developed for use in partial breast reconstruction. The surgical principles underlying these perforator flaps, as well as the vascular anatomy of the respective perforators, have been comprehensively characterised and validated in multiple anatomical and clinical investigations [1, 12, 13, 20].

These techniques provide a minimally invasive and cosmetically favourable option for selected patients and have become a valuable addition to the oncoplastic repertoire.

A crucial component of perforator flap surgery is the identification and preparation of suitable vessels. Pre- and intraoperative imaging of the dominant perforator vessels can provide a decisive aid in flap harvesting. The implementation of effective imaging planning has been demonstrated to result in a reduction in operating times and the prevention of complications arising from the selection of inappropriate vessels. In reconstructive surgery, established methods of imaging perforator vessels include computed tomography angiography (CTA) and magnetic resonance imaging (MRI), which can be used to create three-dimensional reconstructions. [8, 14, 17, 19]. For the most common free flap in breast surgery, the Deep Inferior Epigastric Perforator (DIEP)-Flap, most centres perform a preoperative CTA with a layer thickness of approx. 0.6 mm to describe the topography of the dominant perforators in relation to the umbilicus. The position of the vessels can be confirmed intraoperatively using an audible handheld Doppler ultrasound device.

While computed tomography angiography (CTA) has been considered the gold standard for preoperative perforator mapping due to its spatial resolution and three-dimensional capabilities, colour duplex ultrasound (CDUS) is increasingly recognised as a radiation-free, cost-effective, and real-time alternative. While *Rozen et al.* consider the use of colour Doppler to be of inferior value compared to CTA in planning DIEP-Flap surgery due to the long examination time and interobserver variability, most microsurgeons now use CDUS as a safe and fast method for flap mapping [22].

Visconti et al. described the use of colour duplex ultrasound in the planning and execution of lateral chest wall flaps (LICAP, LTAP, and TDAP). In 95 flaps, the dominant perforator could always be identified using CDUS. There were no flap losses [28].

Building upon previously published studies on the use of colour Doppler ultrasound (CDUS) for perforator assessment, the present work is intended as a complementary contribution with a specific focus on methodological standardisation. We introduce a standardised ultrasound protocol for the evaluation of anterior chest wall perforators and establish a reproducible coordinate system to allow precise topographic mapping of perforator location.

The primary objective is to systematically correlate preoperative CDUS-based localisation and qualitative vessel assessment with intraoperative findings of the vascular pedicle. In addition, spatial distribution patterns of dominant internal and lateral intercostal artery perforators are analysed using a grid-based mapping approach to identify areas of highest anatomical frequency.

The standardisation of pre- and intraoperative perforator imaging may help reduce operative uncertainty and has the potential to improve surgical planning and accessibility of chest wall perforator flaps for a broader range of breast surgeons.

## Materials and methods

### Study design and patient selection

A retrospective single-centre study was conducted at the Breast Centre of the Technical University of Munich between March 2022 and February 2025. All clinical and sonographic examinations, surgical planning, and operative procedures were performed by the same senior breast surgeon. The investigator, serving as senior consultant and head of the breast centre, is formally trained and experienced in both breast ultrasound diagnostics and reconstructive breast surgery. Postoperative follow-up examinations were likewise conducted by the same examiner to ensure consistency in assessment.

While this single-operator design ensured methodological consistency and reduced interobserver variability, it may limit the generalizability of the findings and does not allow assessment of interobserver reliability.

As part of preoperative planning for breast-conserving surgery for invasive or in situ breast cancer, 64 women underwent standardised high-frequency colour Doppler ultrasound (CDUS) mapping of anterior chest wall perforators. All 64 patients were diagnosed with primary breast carcinoma or ductal carcinoma in situ (DCIS) and were candidates for breast-conserving surgery and perforator flap-based reconstruction. All tumours were located in the lower quadrants or the central region of the breast.

Based on the anticipated resection volume, oncoplastic treatment options were discussed with all patients preoperatively, including the possibility of volume replacement using an anterior intercostal artery perforator (AICAP) flap. Depending on breast morphology, breast volume, and individual patient preference, alternative techniques, such as reduction mammoplasty and other oncoplastic approaches, were also considered. The respective advantages and disadvantages, scar patterns, and potential risks of each procedure were thoroughly explained and discussed with the patients. A potential limitation of this study is that the indication and patient selection for AICAP flap reconstruction were determined by a single examiner and surgeon. Patients with an increased anaesthetic risk (e.g., severe cardiac comorbidities) were excluded due to the potentially prolonged operative time associated with the procedure. Additional exclusion criteria for screening and subsequent ICAP-based reconstruction included therapeutic anticoagulation, very low body fat with insufficient inframammary tissue, and conditions associated with a high risk of wound-healing complications, such as morbid obesity (adipositas per magna), poorly controlled type II diabetes mellitus, and severe nicotine abuse. Consequently, the screening cohort and the surgically treated group appeared descriptively similar with regard to tumour characteristics and perioperative risk profile; however, no formal statistical comparison between groups was performed.

**Fig. 1:**
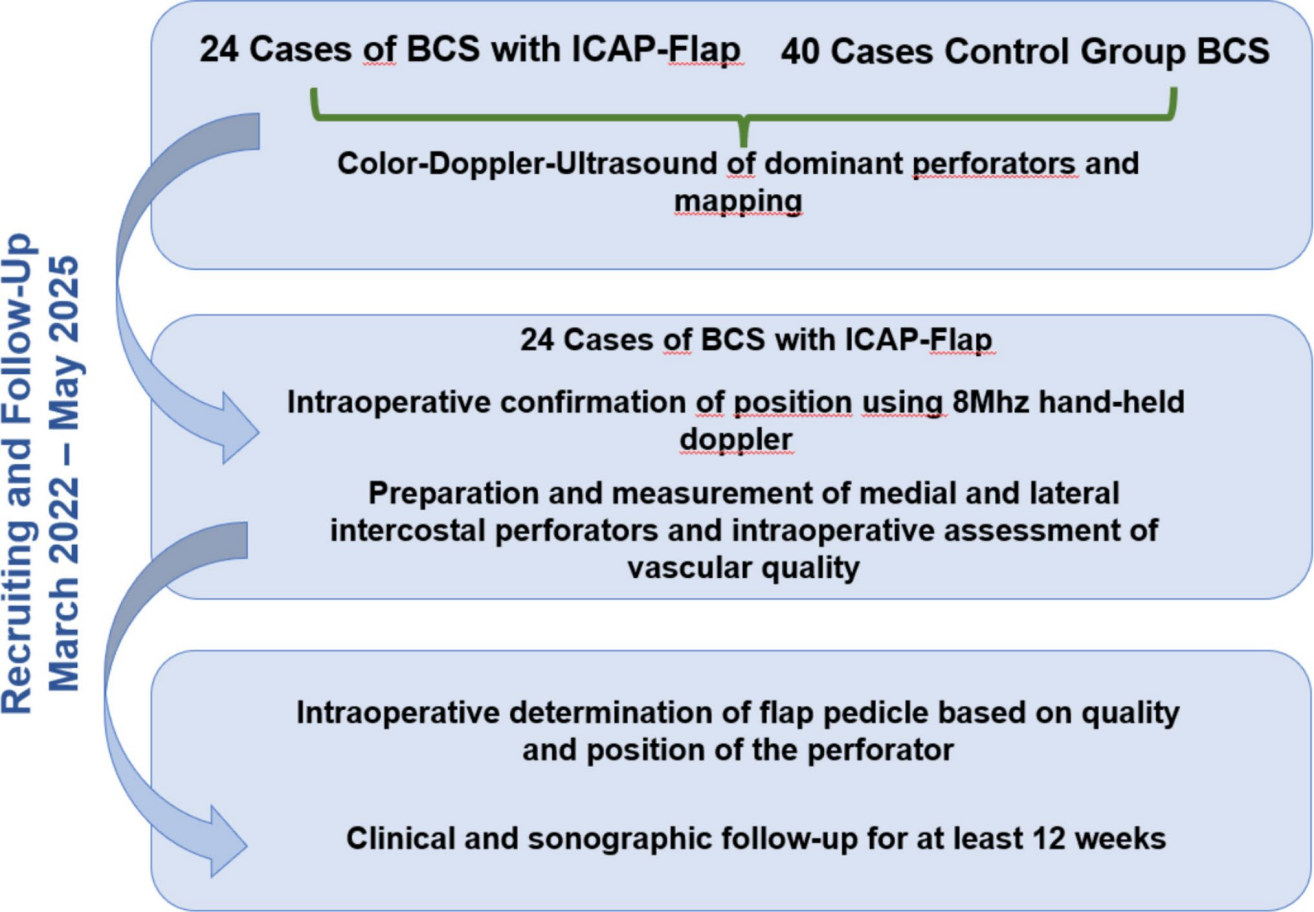
Study flow

Twenty-four patients opted for ICAP flap reconstruction, while 40 underwent alternative oncoplastic techniques (Fig. 1*).*

### Ultrasound protocol

All ultrasound examinations were performed by the same senior breast surgeon with formal training and extensive experience in breast ultrasound diagnostics and oncoplastic breast surgery. Ultrasound examinations were performed using a high-frequency linear transducer (12–18 MHz). To ensure optimal comparability, only two ultrasound systems were used. Preoperative screening was performed with a GE Voluson S8, while the immediate pre- and intraoperative assessments were conducted using a GE Voluson Swift (GE Medical Systems, Düsseldorf, NRW, Germany). Both systems used identical linear transducers and standardised breast sonography presets.

All Doppler settings were standardised according to a predefined protocol optimised for the detection of small-calibre perforator vessels. The pulse repetition frequency (PRF) was adjusted to low-flow settings appropriate for small-calibre vessels (typically 0.5–1.5 kHz). The wall filter was minimised to avoid suppression of low-velocity signals. Colour Doppler gain was initially increased to visualise all detectable vessels and subsequently reduced to minimise background noise and blooming artefacts during diameter measurement.

Depth and focal zone were individually adapted to the thoracic wall anatomy, with the focal zone positioned at the level of the pectoral fascia to optimise visualisation of perforators as they traversed the superficial muscle fascia. As the study focussed on vessel identification and diameter measurement rather than flow quantification, no formal angle correction for velocity calculation was applied. However, care was taken to align the transducer as parallel as possible to the vessel course to optimise Doppler signal detection.

B-mode imaging was used to identify anatomical landmarks, including the sternal border, ribs, and thoracic wall musculature. Using colour Doppler mode, the entire area from the midline to the mid-axillary line and from approximately the fourth intercostal space to approximately 4 cm caudal to the inframammary fold was systematically scanned. The colour Doppler box was initially set wide to ensure full visualisation and then progressively reduced to enable precise measurement.

Vessel diameters were obtained in the longitudinal plane at the point of maximal lumen visualisation, immediately after the vessel traversed the superficial muscle fascia. Each measurement was performed twice, and the mean value was recorded to reduce measurement error.

The dominant perforator was defined as the vessel with the largest visible arterial diameter within the scanned field and a clearly detectable arterial colour Doppler signal. As flow velocity was not quantitatively assessed, dominance was primarily diameter-based and supported by qualitative Doppler signal presence.

Due to the small calibre of the perforators and limited delineation in B-mode imaging, diameter measurements were performed in colour Doppler mode. To minimise blooming artefacts and overestimation, colour gain was carefully reduced after vessel identification until signal spillover beyond the vessel wall was eliminated. Measurements were obtained in the longitudinal plane at the point where the vessel emerged from the superficial muscle fascia. Vessel diameter was measured inner-to-inner using electronic callipers. Each measurement was performed twice, and the mean value was used for analysis.

During the screening examination, anatomical reference lines were marked with the patient in an upright standing position and the arms relaxed alongside the body. The midline, the anterior axillary line, and a tangential line along the lowest point of the inframammary fold were drawn on the skin to establish a standardised coordinate system. The same reference markings were repeated immediately prior to surgery to ensure consistent anatomical orientation.

Ultrasound examinations were performed with the patient in a supine position and the ipsilateral arm abducted approximately 90°. This positioning was used both during outpatient screening and again immediately preoperatively. Care was taken to replicate the planned intraoperative positioning to minimise positional tissue shift and avoid displacement of perforator landmarks.

The identified dominant perforator was displayed centrally within the ultrasound image, and the corresponding skin projection was marked using an indelible marker. Immediately prior to surgery, the marked perforator location was re-verified using a handheld audible Doppler device before sterile preparation.

### Follow-up assessments

Postoperative clinical assessment was performed on days 1–3. In cases of skin-replacement flaps, vascularity was evaluated clinically by assessing colour, temperature, and capillary refill. For volume-replacement flaps, flap vitality was additionally assessed using CDUS.

Routine follow-up visits were scheduled at 2, 6, and 12 weeks postoperatively. During these visits, clinical examination and ultrasound were performed to detect fat necrosis or other perfusion-related complications. Fat necrosis was defined based on imaging characteristics. On ultrasound, fat necrosis typically presents within the flap area as a predominantly complex cystic lesion without internal vascularity on Doppler examination. Over time, fat necrosis may undergo calcification, resulting in characteristic findings on mammography during long-term follow-up. In cases of equivocal findings on ultrasound or mammography, additional evaluation by breast MRI was performed when clinically indicated.

Patients were additionally invited for extended clinical follow-up at 6 months and 1 year to assess long-term outcomes and identify potential radiotherapy-associated changes.

### Anatomy and terminology

The terminology of the intercostal artery perforator flap is used inconsistently in the literature. In our study, we adhere to the anatomical descriptions provided by *Hamdi et al.* and follow the nomenclature of chest wall perforator flaps as defined in the “Gent” Consensus on Perforator Flap Terminology [3]. The *Anterior Intercostal Artery Perforator Flap* is designed as a spindle-shaped flap caudal to the inframammary fold. The vascular supply is derived from the anterior thoracic circulation, primarily the intercostal and superior epigastric vessels. Some authors subdivide the region below the inframammary fold into three distinct zones. Flaps harvested from the medial segment adjacent to the sternal border are referred to as internal anterior intercostal artery perforator (IAICAP) flaps, those based on perforators arising from the central portion are described as middle anterior ICAP (MAICAP) flaps, and flaps supplied by perforators originating from the lateral third are termed lateral anterior ICAP flap (LAICAP) [6, 7]. *Hamdi et al.* described the LICAP flap in 2004 as a lateral chest wall flap based on perforators coursing between the latissimus dorsi muscle and the pectoralis major muscle [13]. Depending on the location of the perforators, lateral anterior ICAP flaps may also partially incorporate perforators arising from this region. Flaps of the lateral chest wall were not included in our study.

In our study, we adopt the above-mentioned nomenclature, which defines AICAP flaps as flaps located below the inframammary fold and further subdivides them into lateral (LAICAP) and internal (IAICAP) types.

### Surgical technique

Flap width was determined using a pinch test with the patient in an upright position; excessive width was avoided to ensure tension-free donor-site closure. Preoperatively and immediately post-tumour excision, flap dimensions and rotation were simulated using surgical gauze (“propeller” manoeuvre), secured at the dominant perforator point to estimate optimal flap length and rotation direction.

Intraoperatively, after tumour resection, the flap was incised and the dominant perforators were identified on the fascial plane and dissected from caudal to cranial. A systematic intraoperative exploration of the entire preoperatively scanned field was performed to identify all accessible perforators within the operative area, ensuring that the dominant vessel selected for flap harvest was confirmed independently of the preoperative marking. Because the predefined scan field matched the anatomical boundaries of the planned flap design, systematic intraoperative exploration covered the complete perforator-bearing area relevant for reconstruction.

The perforator pedicle diameter was measured intraoperatively using a sterile millimetre ruler. The surrounding area was inspected for additional smaller perforators, and vessel quality was assessed qualitatively by pulsatility; however, a systematic search protocol for all potential perforators within the entire anatomical region was not performed. Therefore, diagnostic performance measures, such as sensitivity or negative predictive value, were not calculated. After flap harvest, the tissue was rotated into the resection cavity and secured with absorbable sutures. De-epithelialization was performed when no skin replacement was needed.

### Data acquisition and statistical analysis

Clinical, sonographic, surgical, and follow-up data were extracted from the SAP® hospital information system. Data were analysed anonymously. Statistical analyses were carried out using IBM® SPSS® Statistics Version 26 and Microsoft® Excel® 2019.

Continuous variables are presented as mean (M) ± standard deviation (SD). For non-normally distributed data, results are additionally reported as median and interquartile range (IQR). Normality was assessed using the Shapiro–Wilk test.

Differences between preoperative and intraoperative vessel diameters were evaluated using paired-samples *t *tests, as the distribution of paired differences met the assumption of normality. Ninety-five percent confidence intervals (95% CI) were calculated for mean differences. Confidence intervals were calculated to provide an estimate of the precision of the observed differences. Effect sizes were determined using Cohen’s *d*.

Comparisons between right- and left-sided vessel diameters were performed using the Mann–Whitney U test, because the data were not normally distributed and group sizes were unequal. All tests were two-tailed, and a p value < .05 was considered statistically significant. As analyses were primarily exploratory, no formal adjustment for multiple comparisons was applied and results should be interpreted accordingly.

For spatial distribution analysis, perforator coordinates were referenced to predefined anatomical landmarks, including the midline, the inframammary fold (IMF), and the anterior axillary line, which had been identified and marked in the upright position. The inframammary fold was identified and marked with the patient in the upright position during the preoperative examination and served as the horizontal reference axis for coordinate measurements. Distances were measured in centimetres using a measuring tape as linear projections relative to the predefined reference axes. For internal perforators, lateral distances from the midline were recorded as positive values. For lateral perforators, distances medial to the anterior axillary line were defined as positive and lateral positions as negative. Positions inferior to the IMF were recorded as negative values according to the predefined axis convention. No normalisation to breast size was performed, as the coordinate system was based on fixed anatomical landmarks to ensure clinical reproducibility.

Coordinates were rounded to the nearest centimetre and aggregated using a two-dimensional grid-based binning approach (1-cm intervals). No smoothing or kernel density estimation was applied in order to preserve the original distribution of perforator locations. The number of dominant perforators per grid cell was calculated and visualised using a bubble plot, with marker size proportional to the frequency. All 64 mapped patients were included in the spatial distribution analysis, irrespective of whether AICAP flap reconstruction was ultimately performed.

The primary endpoint was the agreement between the dominant perforator identified by preoperative CDUS and the dominant perforator confirmed intraoperatively during flap dissection.

As a secondary endpoint, spatial accuracy was quantified by calculating the Euclidean distance (cm) between the preoperative skin marking and the intraoperative perforator emergence point.

Distances were derived from two-dimensional coordinates referenced to predefined anatomical landmarks. Localisation deviation was reported as mean ± SD or median (IQR), depending on distribution. Frequency distributions were illustrated using histograms to assess dispersion and potential outliers.

Because intraoperative exploration was restricted to the predefined operative field and not intended as a comprehensive anatomical search, diagnostic performance metrics, such as sensitivity or negative predictive value, were not calculated.

### Ethical approval

The study was reviewed and approved by the Ethics Committee of the Technical University of Munich (2023-113-S-SR). Due to the retrospective nature of the study, the requirement for informed consent was waived by the ethics committee.

## Results:

### Patient characteristics and follow-up

A total of 64 patients underwent colour Doppler ultrasound (CDUS) as part of their preoperative planning for breast-conserving surgery. Of these, 40 patients received breast-conserving surgery with conventional oncoplastic techniques, while in 24 patients, reconstruction of the defect was achieved using an anterior ICAP flap.

Baseline characteristics of patients undergoing AICAP reconstruction and those treated with alternative oncoplastic techniques are summarised in Table 1. Demographic variables and tumour-related parameters, including BMI, clinical T-stage distribution, tumour localisation, smoking status, and prevalence of type II diabetes, were descriptively similar between groups. No formal statistical comparison was performed, as analyses were exploratory and not powered for between-group inference.

**Table 1:** Baseline demographic and tumour characteristics of patients undergoing AICAP flap reconstruction compared with patients receiving alternative oncoplastic procedures. Data are presented as mean ± SD or n (%). Percentages refer to valid cases. No formal statistical comparison between groups was performed

Variable	Overall (*n*=64)	AICAP (*n*=24)	Non-AICAP (*n*=40)
BMI (mean ± SD)	25.89 ± 4.35	26.08 ± 4.62	25.77 ± 4.23
Clinical T-Stadium, n (%)			
cTis	13 (20,3%)	5 (20,8%)	8 (20%)
cT1	24 (37,5%)	8 (33,3%)	16 (40%)
cT2	27 (42,2%)	11 (45,8%)	16 (40%)
Tumour localisation, n (%)			
Central	20 (31,25%)	8 (33,3%)	12 (30%)
Lower inner quadrant	24 (37,5%)	9 (37,5%)	15 (37,5%)
Lower outer quadrant	20 (31,25%)	7 (29,2%)	13 (32,5%)
Smokers, n (%)	12 (18,8%)	4 (16,7%)	8 (20%)
Diabetes mellitus, n (%)	4 (6,3%)	1 (4,2%)	3 (7,5%)

The mean age was 54.8 years (range: 27–82). In 38 cases, perforators of the right breast were examined, and in 26 cases, those of the left breast.

The mean operative time for AICAP flap procedures was 99 min (SD 5.17; range 51–160). The operative time was measured from the initial skin incision to completion of skin closure. The entire tumour resection and, if applicable, intervention on the axillary lymph nodes were performed within this time interval. Operative times for alternative oncoplastic procedures were not analysed for comparison. In all cases, adjuvant radiotherapy was recommended following breast-conserving surgery. The mean follow-up period was 29 weeks (SD 3.59; range 12–64 weeks). No perfusion-related flap complications (e.g., skin necrosis or liponecrosis) were observed during the immediate postoperative period or within the first 12 weeks. A wound-healing complication in the inframammary fold was observed in one patient and could be resolved with conservative management followed by secondary closure. Eleven of 24 reconstructed patients (46%) completed a 6-month follow-up visit, and five patients (21%) reached at least 56 weeks of follow-up. In one patient, 52 weeks after surgery and adjuvant radiotherapy, imaging revealed signs of fat necrosis despite an unremarkable skin island of the flap. Ultrasound and MRI demonstrated a complex cystic component measuring approximately 2.5 × 2 cm at the dorsal margin in the middle third of the LAICAP flap, without signs of internal vascularization or calcification. The patient reported no associated symptoms, and no surgical revision has been required to date. A follow-up examination is scheduled in three months. No additional cases of clinically relevant fat necrosis were observed during the available follow-up period.

In all 24 cases, an R0 resection with tumour-free margins was achieved during the primary procedure, and therefore, no re-excisions were required.

### Perforator characteristics

A paired-samples t test was conducted to compare preoperative and intraoperative vessel diameters. Key perforator measurements and localisation parameters are summarised in Table 2.

**Table 2:** Patient characteristics, preoperative perforator measurements obtained by colour Doppler ultrasound (CDUS), and intraoperative findings in patients undergoing anterior intercostal artery perforator (AICAP) flap reconstruction compared with patients treated with alternative oncoplastic techniques. Continuous variables are presented as mean ± standard deviation (SD) or mean (range) as indicated

Parameter	Overall (*n* = 64)	AICAP (*n* = 24)	Non-AICAP (*n* = 40)
Age (years), mean (range)	54.8 (27–82)	55.6 (37–82)	54.3 (27–78)
Right vs. left breast, n	38 vs. 26	15 vs. 9	23 vs. 17
LAICAP diameter (CDUS, mm), mean ± SD	2.19 ± 0.50	2.08 ± 0.30	2.25 ± 0.60
IAICAP diameter (CDUS, mm), mean ± SD	1.81 ± 0.40	1.61 ± 0.30	1.93 ± 0.40
Intraoperative LAICAP diameter (mm), mean ± SD	—	1.69 ± 0.29	—
Intraoperative IAICAP diameter (mm), mean ± SD	—	1.10 ± 0.28	—
IAICAP distance to midline (cm), preoperative mean ± SD	3.63 ± 0.90	3.44 ± 0.60	3.73 ± 1.00
LAICAP distance to anterior axillary line (cm), preoperative mean ± SD^a^	−0.53 ± 1.20	−0.67 ± 1.30	−0.46 ± 1.10
Euclidean localization deviation IAICAP (cm) mean ± SD	—	0.25 ± 0.10	
Euclidean localization deviation LAICAP (cm) median (IQR)		0.20 (IQR 0.03–0.20)	
Selected flap design (LAICAP vs IAICAP), n	—	20 vs. 4	—
Concordance between CDUS and intraoperative findings (%)	—	100%	—

^a^Negative values indicate positions lateral to the anterior axillary line

For the lateral perforator, the preoperative diameter (M = 2.08 mm, SD = 0.31) was significantly larger than the intraoperative diameter (M = 1.69 mm, SD = 0.29). The mean difference was 0.39 mm (95% CI [0.29, 0.49]). This difference was statistically significant, *t*(23) = 8.03, *p* < .001, indicating a substantial reduction in vessel diameter intraoperatively. The effect size was large (Cohen’s *d* = 1.64).

Similarly, for the internal perforators, the preoperative diameter (M = 1.61 mm, SD = 0.26) was significantly larger than the intraoperative diameter (M = 1.10 mm, SD = 0.28). The mean difference was 0.513 mm (95% CI [0.38, 0.65]). This reduction was statistically significant, *t*(23) = 7.89, *p* < .001, with a large effect size (Cohen’s *d* = 1.61).

Agreement between CDUS and intraoperative diameter measurements was further evaluated using Bland–Altman analysis, which demonstrated a systematic overestimation of vessel diameter by CDUS. For the internal perforators (IAICAP), the mean bias was 0.54 mm, with 95% limits of agreement ranging from 0.01 mm to 1.07 mm. For the lateral perforators (LAICAP), the mean bias was 0.39 mm, with 95% limits of agreement ranging from −0.08 to 0.86 mm.

Visual inspection of the Bland–Altman plots revealed no evidence of relevant proportional bias, as differences were evenly distributed across the measurement range.

**Fig. 2:**
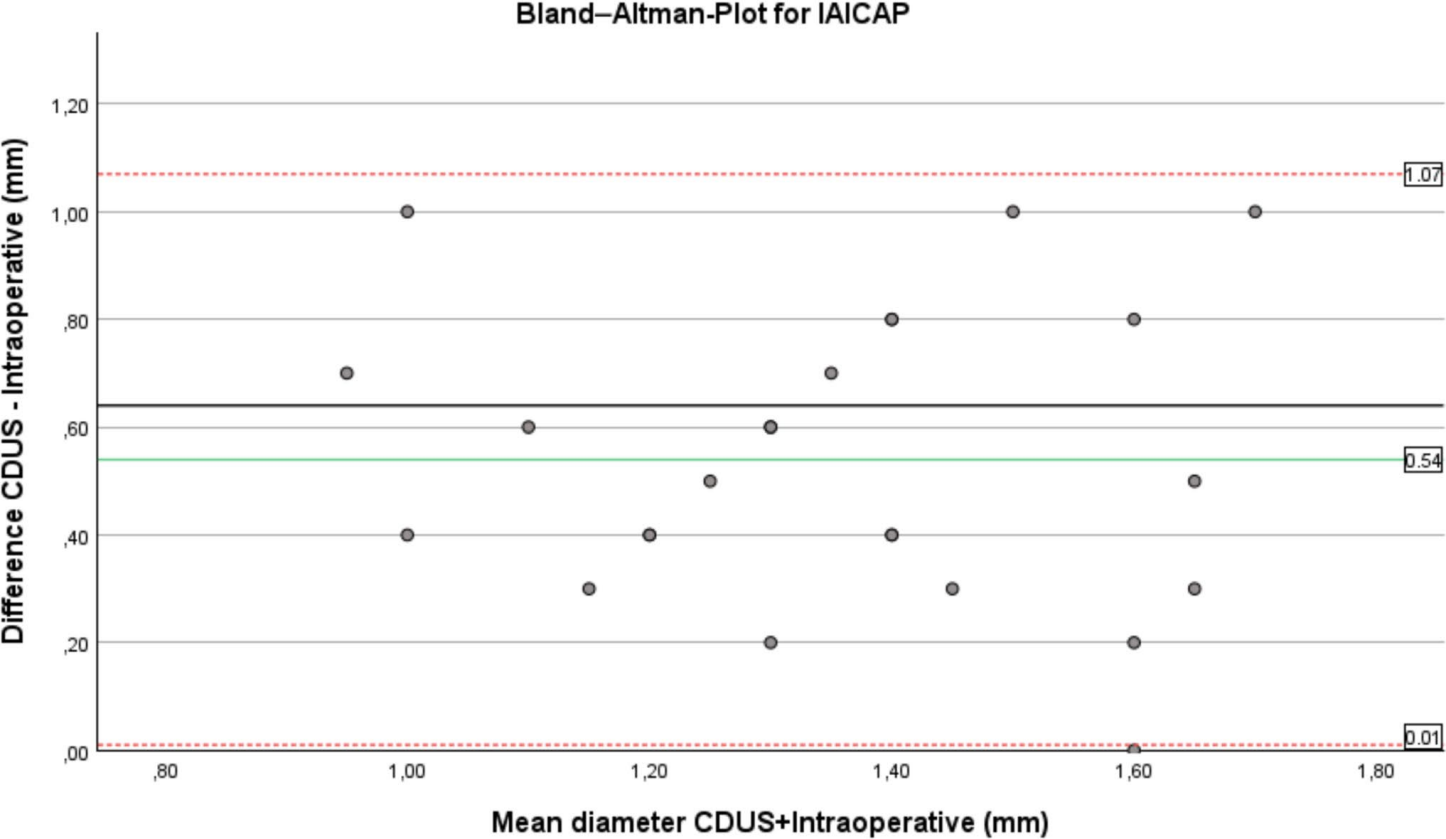
Bland–Altman plot comparing preoperative CDUS and intraoperative measurements for IAICAP (bias 0.54 mm; 95% LoA 0.01–1.07 mm)

**Fig. 3:**
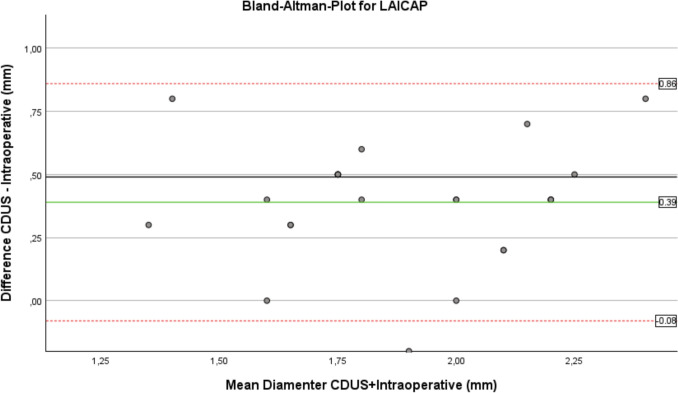
Bland–Altman plot comparing preoperative CDUS and intraoperative measurements for LAICAP (bias 0.39 mm; 95% LoA −0.08–0.86 mm)

Although paired t tests confirmed statistically significant differences between preoperative and intraoperative diameters, Bland–Altman analysis demonstrates that CDUS consistently measures larger vessel diameters, with a mean overestimation of approximately 0.4–0.5 mm (Figs. 2, 3).

38 right-sided and 26 left-sided vessels were analysed. The mean diameter of lateral perforators on the right side was *M* = 2.21 mm (SD = 0.57), whereas on the left side, it was *M* = 2.16 mm (SD = 0.44). For the internal perforators, the mean diameter on the right side was *M* = 1.77 mm (SD = 0.43), compared to *M* = 1.87 mm (SD = 0.39) on the left side.

To compare vessel diameters between the right and left sides, Mann–Whitney U tests were performed, because the data were not normally distributed and the groups represented independent observations.

For the LAICAP, no significant difference in vessel diameter was observed between the right *U* = 472.50, *z* = −0.296, *p* = .768. Similarly, for the IAICAP, vessel diameter did not differ significantly between the right and left sides, *U* = 585.50, *z* = 1.256, *p* = .209. These findings indicate that vessel diameter did not differ significantly between the right and left sides.

**Fig. 4:**
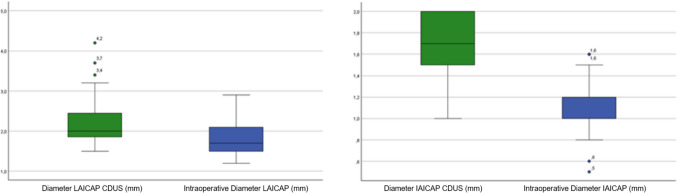
Comparison of perforator diameters measured preoperatively by CDUS and intraoperatively

When comparing intraoperative diameters between perforator types, lateral perforators (M = 1.69 mm, SD = 0.29) were significantly larger than internal perforators (M = 1.10 mm, SD = 0.28). The mean difference was 0.600 mm (95% CI [0.483, 0.717]). This difference was statistically significant, t(23) = 10.63, p < 0.001 (Fig. 4).

### Concordance and localisation accuracy

In all 24 surgical cases, the dominant perforator identified by preoperative CDUS was confirmed intraoperatively, resulting in complete agreement within the predefined operative field (24/24 cases). This concordance was based on systematic intraoperative exploration of the entire preoperatively scanned operative field.

Localisation accuracy was quantified by calculating the Euclidean distance between the preoperative skin marking and the intraoperative perforator emergence point.

For IAICAP perforators, the mean localisation deviation was 0.25 ± 0.10 cm.

For LAICAP perforators, the median deviation was 0.20 cm (IQR 0.03–0.20 cm). As localisation deviation for LAICAP perforators was not normally distributed, results are presented as median and interquartile range.

**Fig. 5:**
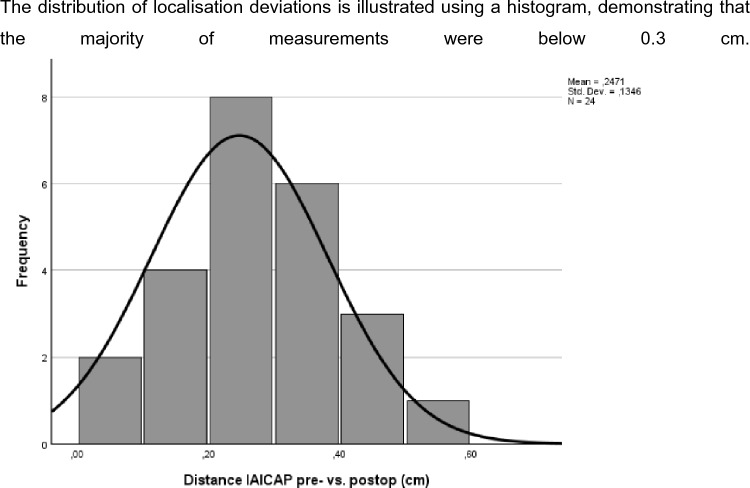
Histogram of Euclidean localisation deviation (cm) between preoperative CDUS skin marking and intraoperative perforator emergence point for IAICAP perforators (n = 24)

**Fig. 6:**
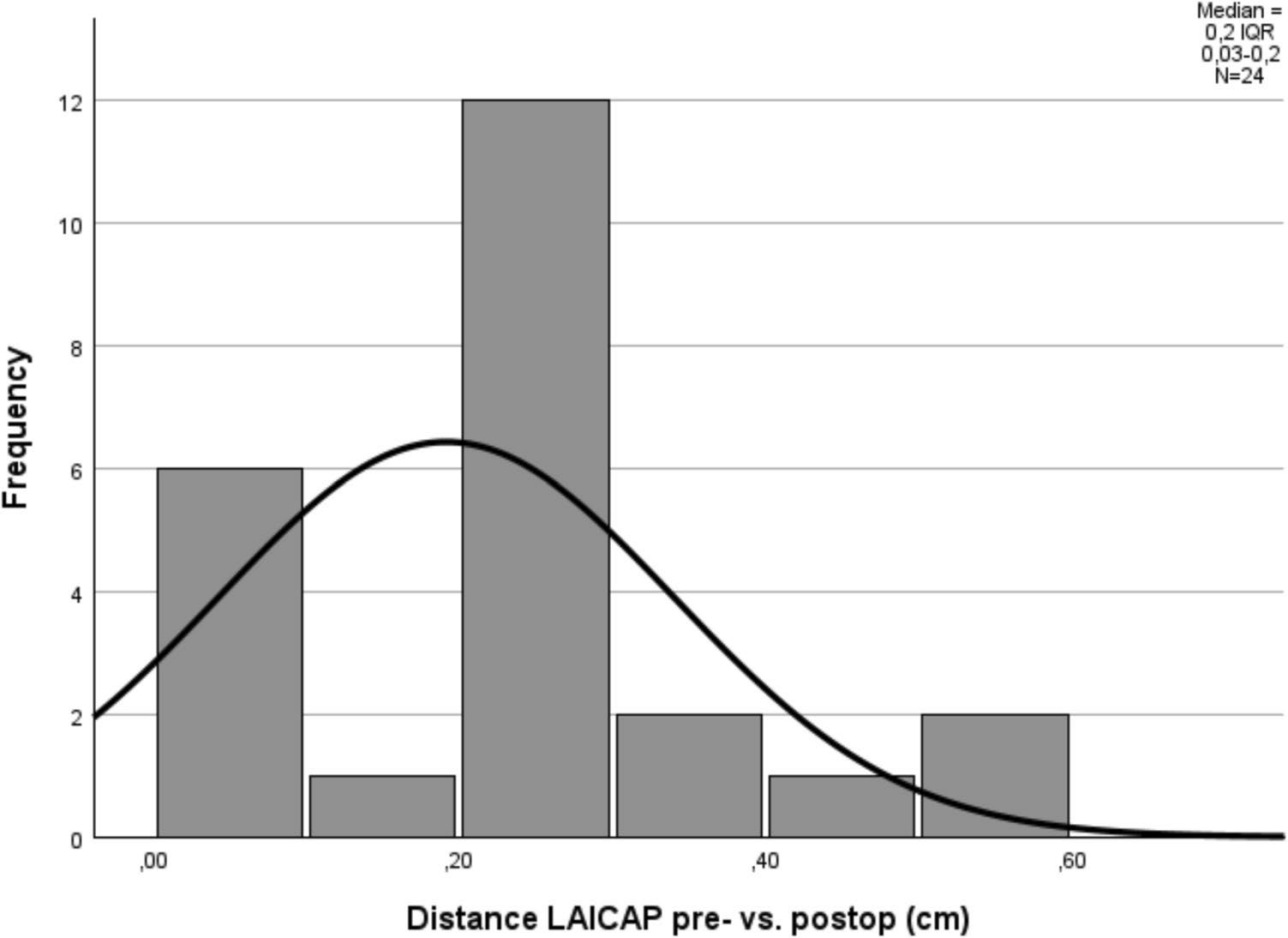
Histogram of Euclidean localisation deviation (cm) between preoperative CDUS skin marking and intraoperative perforator emergence point for LAICAP perforators (n = 24)

The distribution of localisation deviations is illustrated using a histogram, demonstrating that the majority of measurements were below 0.3 cm (Figs. 5, 6).

### Intraoperative decision-making and flap selection

Intraoperative dissection and measurement of both dominant internal and lateral perforators were performed in 24 patients. All cases involved patients with either invasive breast cancer or ductal carcinoma in situ. Preoperative imaging and the assessment of tumour size relative to breast volume permitted breast-conserving surgery. In all procedures, intraoperative ultrasound was used to evaluate the resection margins, complemented by specimen radiography to confirm complete tumour removal. Histopathological evaluation confirmed tumour-free in all cases, and therefore, no re-excisions were required.

Intraoperative selection of the pedicle (internal vs. lateral perforator) was based on vessel quality and anatomical defect location. For defects located in the lower quadrants or central breast, the decision was guided by which pedicle would offer the optimal reconstructive outcome. Vessel assessment included intraoperative evaluation of diameter and arterial pulsatility. Furthermore, the presence of adjacent smaller perforators in close proximity to the dominant vessel was documented, assuming they did not compromise tension-free flap rotation. An average of 2.04 (range: 1–3) additional lateral perforators and 1.4 (range: 1–3) additional internal perforators were identified per patient in the relevant operative fields.

Preoperative planning identified the presumed optimal pedicle in all patients. The surgical plan was adhered to in 23 of 24 cases. In one case, intraoperative finding necessitated conversion from an IAICAP to an LAICAP flap due to insufficient calibre of the dominant internal perforator.

**Fig. 7:**
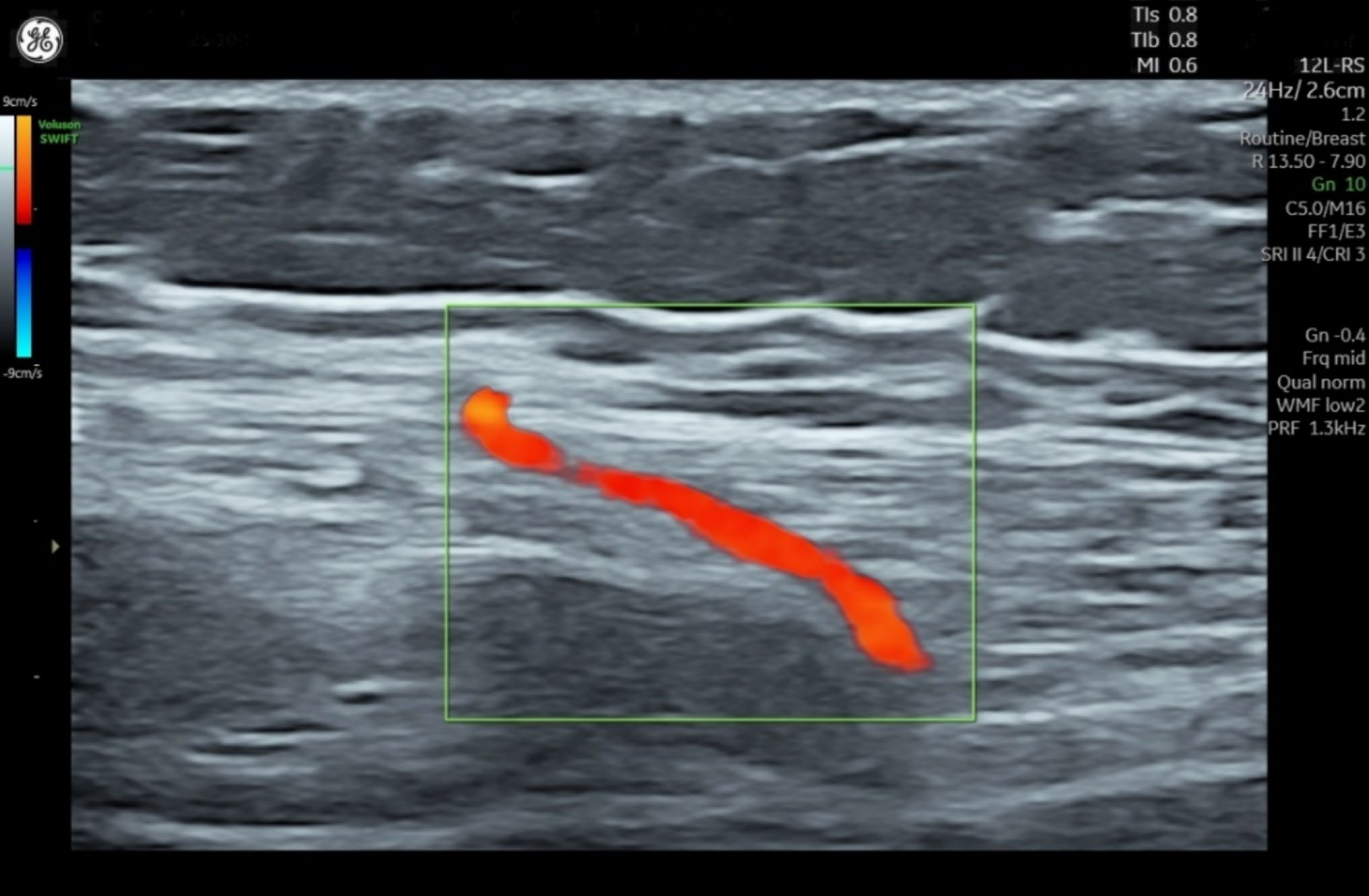
Course of an internal intercostal perforator. The perforator originates from the sixth intercostal space and traverses the thoracic wall musculature towards the superficial fascia

Ultimately, 20 LAICAP and 4 IAICAP flaps were performed (Fig. 7).

### Spatial distribution of perforators

To facilitate standardised localisation of perforators, a coordinate system was established using the patients’ midline, a tangential line along the inframammary fold (IMF), and the anterior axillary line as reference points. During ultrasonography, the transducer was oriented vertically to display the dominant perforator centrally on the monitor. The perforator location was marked on the patient’s skin, and distances to the midline, IMF, and anterior axillary line were measured with a tape measure.

Dominant internal perforators were located at a mean distance of 3.63 cm (range: 1.5–6 cm) lateral to the patient’s midline and 1.32 cm inferior to the IMF. Dominant lateral perforators were situated on average 0.53 cm (range: –2 to +2 cm) medial to the anterior axillary line and 0.95 cm inferior to the IMF (range: –3.5 to +2 cm).

**Fig. 8:**
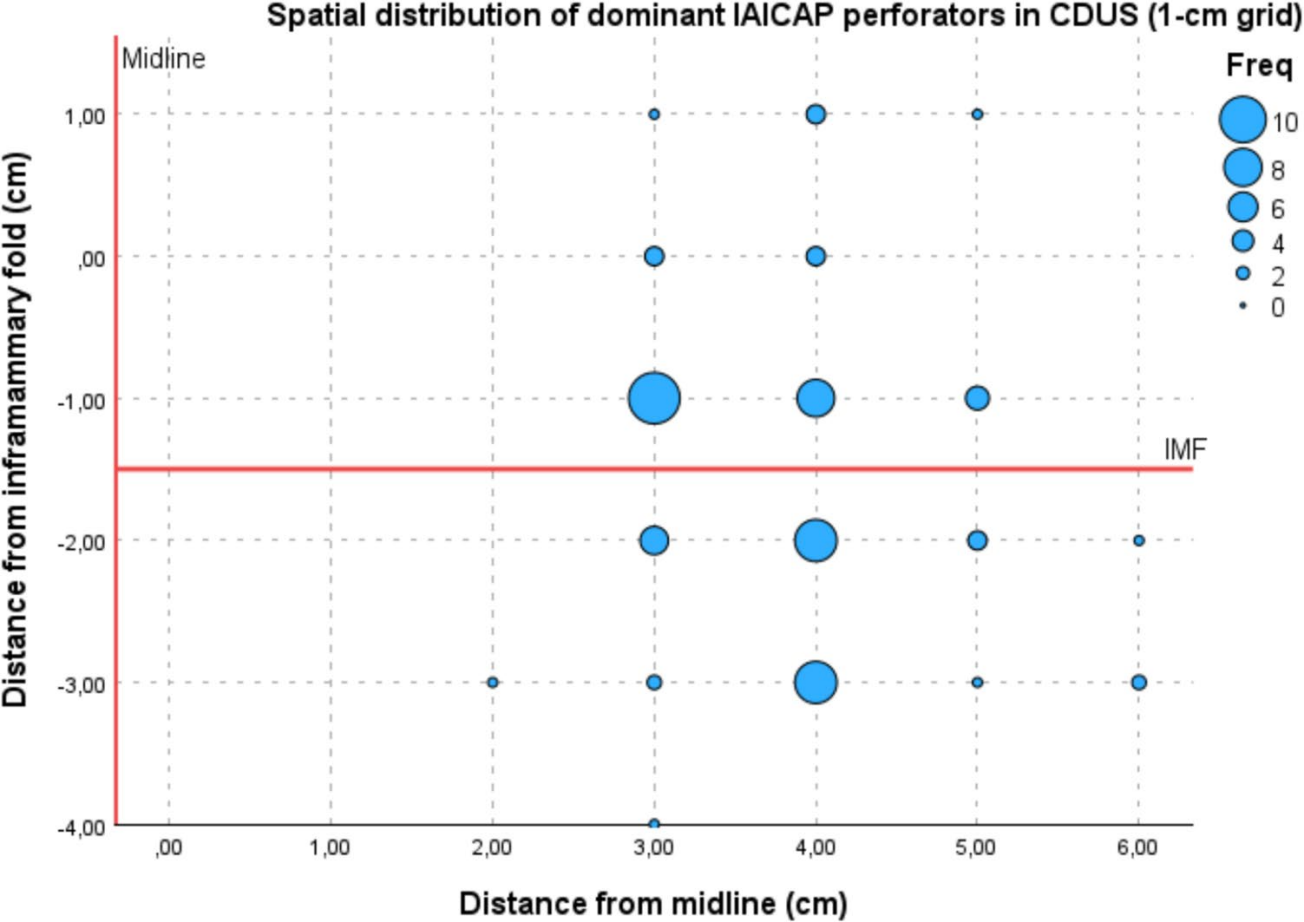
Spatial distribution of dominant IAICAP perforators. Grid-based density bubble plot (1-cm intervals) of dominant IAICAP perforators identified by CDUS. Marker size reflects perforator frequency per grid cell. The vertical red line indicates the midline (x = 0) and the horizontal red line indicates the inframammary fold (IMF; y = 0)

**Fig. 9:**
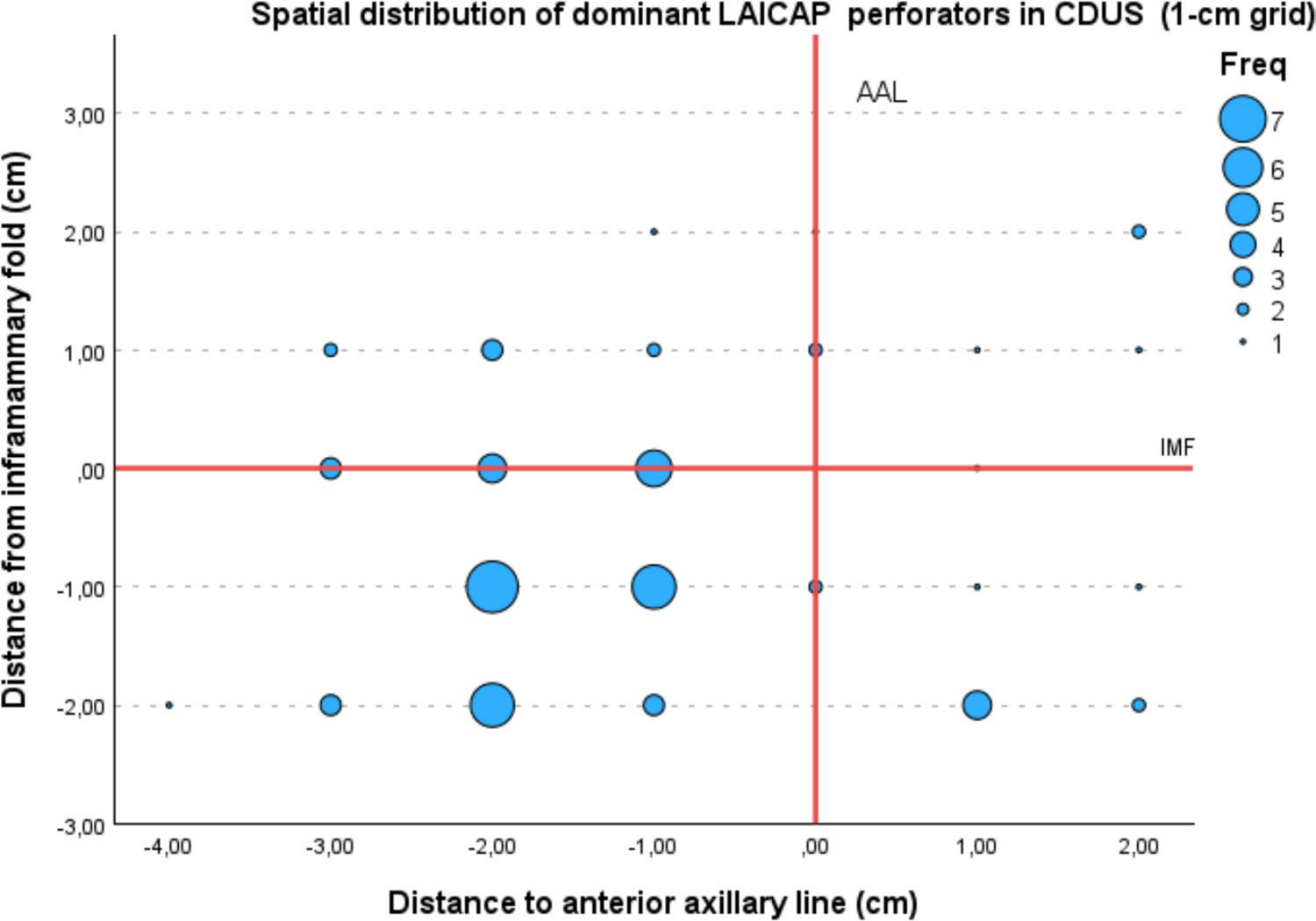
Spatial distribution of dominant LAICAP perforators. Grid-based density bubble plot (1-cm intervals) of dominant LAICAP perforators identified by CDUS. Marker size reflects perforator frequency per grid cell. The vertical red line indicates the anterior axillary line (AAL; x = 0) and the horizontal red line indicates the inframammary fold (IMF; y = 0)

Spatial distribution was further illustrated using a grid-based density bubble plot. After rounding coordinates to 1-cm intervals, perforator frequency per grid cell was calculated. The resulting visualisation demonstrated clear clustering patterns for both internal and lateral perforators, with distinct high-density areas corresponding to the most frequent anatomical locations. The highest density of IAICAP perforators was observed at x = 3 cm and y = −1 cm (n = 10), whereas the highest density of LAICAP perforators was observed at x = −1 cm and y = −2 cm (n = 7). The zero reference lines corresponding to the IMF, midline, and anterior axillary line were highlighted in the graphical representation for orientation (Figs. 8 and 9*).*

## Discussion

Over the past three decades, increasing knowledge of local chest wall perforators has fostered the development of innovative techniques in oncological breast surgery. Advances in microsurgery have enabled the use of increasingly smaller-calibre vessels for perforator flaps, achieving favourable aesthetic outcomes without compromising oncological safety. The dissection of small vessels may appear discouraging to less experienced microsurgeons; therefore, thorough preoperative planning with imaging of the vascular supply may help improve surgical safety and reduce the risk of complications.

Ultrasound is an essential tool in breast surgery, providing diagnostic value before surgery and guidance during surgery. It represents an important component of contemporary oncological breast care. Intraoperative ultrasound is safe, non-invasive, cost-effective, and has been shown to significantly reduce re-excision rates for positive margins [2, 4, 15, 24]. According to current evidence, the AGO Breast guidelines (Arbeitsgemeinschaft für gynäkologisches Onkologie) recommend intraoperative ultrasound for palpable as well as non-palpable tumours [27]. Breast surgeons who routinely perform breast ultrasound can rapidly learn to identify perforator vessels using CDUS and integrate this into their operative workflow.

The combined value of intraoperative ultrasound (IOUS) and CDUS-based planning of chest wall perforator flaps was highlighted by *Ferrucci et al.* [10]. The authors demonstrated the significant oncological benefit of intraoperative ultrasound in increasing the rate of tumour-free resection margins, while simultaneously highlighting the reliable planning of chest wall perforator flaps using CDUS. Perfusion quality in this study was evaluated with pulsed-wave Doppler. Although this approach is promising, acceptable ranges for perforator flow velocity remain undefined. Following flap elevation and concentration of perfusion to one or two dominant vessels, an initial rise in flow velocity is expected, followed by gradual normalisation. Larger cohort studies are needed to establish reference values and to evaluate correlations between flap dimensions, vessel volumes, and flow characteristics.

In the preoperative planning of free perforator flaps, such as the Deep Inferior Epigastric Artery Perforator (DIEP) Flap, the Superficial Inferior Epigastric Artery Perforator (SIEAP) Flap, or the Profunda Artery Perforator (PAP) Flap, Computed Tomography Angiography (CTA) or Magnetic Resonance Angiography (MRA) is standard practice in most centres for visualising the vascular anatomy [14, 19]. Compared with CDUS, CTA and MRA offer a broader assessment of the vascular network, enabling visualisation of perforator course, contralateral vessels, and anatomical variations. However, their disadvantages include radiation exposure, contrast administration, higher cost, and limited availability relative to ultrasound. Moreover, CTA depicts vessel anatomy only in the position of imaging—changes in patient positioning during surgery may reduce intraoperative accuracy, whereas ultrasound allows direct real-time verification. In the German DRG (Diagnosis Related Groups) reimbursement system, chest wall perforator flaps are scarcely represented in the context of oncoplastic breast surgery. As a result, additional imaging modalities, such as CTA or MRA, place further strain on the budget, making cost-efficient surgical management difficult to achieve.

A systematic review and meta-analysis by *Teunis et al.* revealed that the use of preoperative CTA rather than preoperative Doppler ultrasound in DIEP flaps was associated with a significant reduction in partial necrosis and complete flap loss [26]. In addition to reducing risk, the CTA method also provides a high level of prediction regarding the location and quality of perforators in DIEP flaps [21]. However, it should be noted that the studies in the above-mentioned meta-analysis compare CTA with Doppler ultrasound, rather than with high-frequency CDUS. The article does not clarify whether audible handheld Doppler was used exclusively. While acoustic Doppler is able to detect vessels intraoperatively at the intended surgical site, it is limited by its inability to assess vessel diameter, anatomical course, and the presence of accompanying vessels.

A further study in 53 patients undergoing anterior thigh flap reconstruction compared CTA, CDUS, and handheld Doppler. CDUS demonstrated the highest specificity, sensitivity, and overall accuracy. Consistent with our findings, preoperative CDUS correlated perfectly with intraoperative perforator identification [11].

Data on preoperative imaging of thoracic wall perforators remain limited. *Brandt et al.* investigated the visibility of the perforators of the internal mammary, lateral intercostal and dorsal intercostal arteries in 150 patients using CTA [5]. Perforators of the internal mammary artery were visible in 99% of cases, and lateral and dorsal intercostal perforators in 94% and 87%, respectively. Their study aimed to identify perforators suitable for perforator-to-perforator flaps, and did not compare preoperative imaging with intraoperative findings. In contrast, our study preoperatively identified the dominant perforator of largest diameter and additionally documented nearby perforators intraoperatively. Using this approach, we observed an average of 2.04 additional LAICAP and 1.4 additional IAICAP vessels. Vessel diameters reported by CTA were smaller than our CDUS measurements; however, intraoperative measurements were closer to CTA values (LICAP: CDUS 2.19 mm vs. intraoperative 1.7 mm vs. CTA 1.26 mm). This discrepancy likely reflects the flow-based visualisation and lower spatial resolution of Doppler ultrasound. Despite optimised Doppler gain settings, the phenomenon of the “blooming artefact” may explain the difference between the preoperatively and intraoperatively measured vessel diameters in our study.

A subsequent study assessed anterior intercostal artery perforators (AICAP) in a small cohort of 10 clinical cases and 14 cadaveric dissections [6]. Dissection on the anatomical models revealed relatively small vessel diameters, averaging 0.42 mm, with lateral vessels consistently larger than internal ones. Measurements were performed only on cadavers, not in clinical cases, and preoperative assessment was limited to acoustic handheld Doppler. It is likely that the absence of perfusion in cadaveric tissue contributed to smaller measured diameters compared with those observed in clinical practice and in our study.

Another study evaluated the visualisation of lateral chest wall perforators using standard preoperative breast magnetic resonance imaging (MRI). In 50 patients, it was assessed whether lateral thoracic wall perforators could be identified on routine preoperative MRI. The lateral thoracic artery was successfully visualised bilaterally in 48 cases, and in approximately two-thirds of these communicating with perforating intercostal vessels [16]. In contrast to this study, *Salgarello et al.* investigated the use of ultrasound for the planning of lateral chest wall perforator flaps [23]. In this study, the dominant perforators were identified by ultrasound in 11 TDAP and 41 LICAP flaps. In two cases, no perforator could be visualised, and a latissimus dorsi flap was performed instead. Compared with our study, less emphasis was placed on vessel quality and precise anatomical localisation. Importantly, both studies focussed exclusively on lateral perforators.

*Tashiro et al*. demonstrated the utility of CDUS in reliably identifying lower-extremity perforators for perforator-to-perforator reconstruction, also assessing blood flow velocity—though the clinical significance remains unclear [25]. At our institution, pulse-wave Doppler assessment in free perforator flaps is under investigation; future applications may include guiding perforator selection and early detection of hypoperfusion.

High-frequency CDUS enabled reproducible preoperative localisation of dominant IAICAP and LAICAP perforators and showed high agreement with intraoperative confirmation in this cohort. The present study combines a standardised scan protocol, coordinate-based mapping, and quantitative agreement analysis for anterior intercostal perforators. Further prospective studies are needed to determine whether CDUS-guided planning improves operative efficiency or clinical outcomes compared with standard practice.

### Limitations

This study has several limitations that should be acknowledged. The retrospective single-centre design, the relatively small sample size, and the fact that all sonographic examinations were performed by a single surgeon are important limitations. Although the single-operator design ensured methodological consistency, it precludes assessment of interobserver reliability and limits conclusions regarding the general applicability of the standardised ultrasound protocol. The possibility of interobserver variation cannot be excluded. These factors may limit the generalizability of our findings and introduce potential bias. Future research would benefit from a prospective, multicentre approach, incorporation of comparative imaging modalities such as CTA, and assessment by multiple examiners to account for interobserver variability. Such studies could not only confirm our observations but also provide more robust evidence to support the clinical relevance of our findings.

## Data Availability

All data and materials are available upon reasonable request.
